# High Protein Diet and Metabolic Plasticity in Non-Alcoholic Fatty Liver Disease: Myths and Truths

**DOI:** 10.3390/nu11122985

**Published:** 2019-12-06

**Authors:** Francesco De Chiara, Cynthia Ureta Checcllo, Javier Ramón Azcón

**Affiliations:** 1 Biosensors for Bioengineering Group, Institute for Bioengineering of Catalonia (IBEC), The Barcelona Institute of Science and Technology (BIST), Baldiri I Reixac, 10-12, 08028 Barcelona, Spain; jramon@ibecbarcelona.eu; 2 Faculty of Medicine and Health Sciences, University of Barcelona (UB), Gran Via de les Corts Catalanes, 585, 08007 Barcelona, Spain; cynthiur@gmail.com

**Keywords:** NAFLD, NASH, high protein diet, low carbohydrates, physical activity

## Abstract

Non-alcoholic fatty liver disease (NAFLD) is characterized by lipid accumulation within the liver affecting 1 in 4 people worldwide. As the new silent killer of the twenty-first century, NAFLD impacts on both the request and the availability of new liver donors. The liver is the first line of defense against endogenous and exogenous metabolites and toxins. It also retains the ability to switch between different metabolic pathways according to food type and availability. This ability becomes a disadvantage in obesogenic societies where most people choose a diet based on fats and carbohydrates while ignoring vitamins and fiber. The chronic exposure to fats and carbohydrates induces dramatic changes in the liver zonation and triggers the development of insulin resistance. Common believes on NAFLD and different diets are based either on epidemiological studies, or meta-analysis, which are not controlled evidences; in most of the cases, they are biased on test-subject type and their lifestyles. The highest success in reverting NAFLD can be attributed to diets based on high protein instead of carbohydrates. In this review, we discuss the impact of NAFLD on body metabolic plasticity. We also present a detailed analysis of the most recent studies that evaluate high-protein diets in NAFLD with a special focus on the liver and the skeletal muscle protein metabolisms.

## 1. Introduction

Non-alcoholic fatty liver disease (NAFLD) is often defined as the liver manifestation of the metabolic syndrome (MetS), identified by the presence of three or more following features: (i) large waist circumference; (ii) high triglycerides in the blood; (iii) low high-density lipoprotein cholesterol; (iv) hypertension [1]. Patients with MetS are at higher risk of developing coronary heart disease, stroke and diabetes mellitus type 2 (T2DM). 

**Figure 1:**
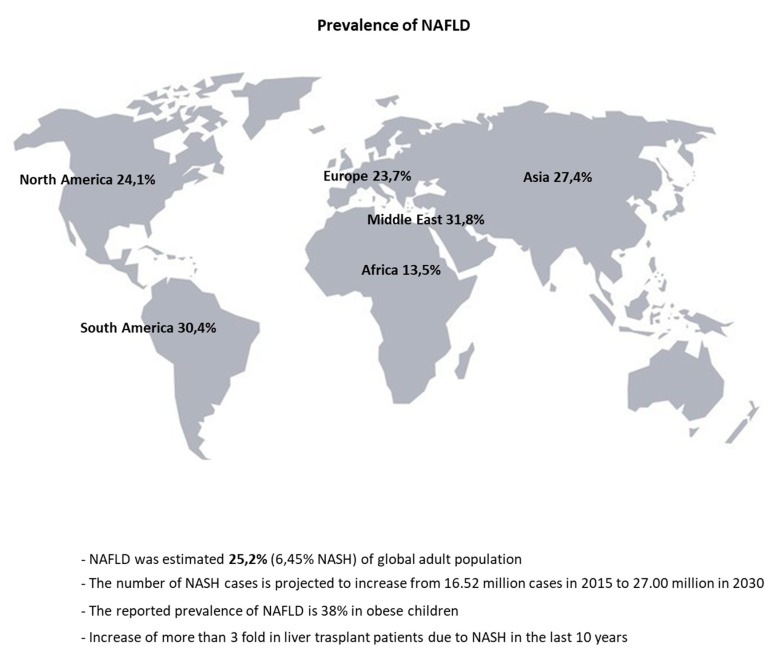
Worldwide distribution of Non-alcoholic fatty liver disease (NAFLD).

The past few years have witnessed the dramatic rise in global NAFLD prevalence in correlation with other non-communicable disease such as obesity and diabetes [2]. Over 25% of the adult population in the world suffers from NAFLD, even though it is underdiagnosed due to its non-specific symptoms [3,4,5] (Figure 1). In obese children, the reported NAFLD prevalence is 38%, it is expected to become the main cause for liver failure. This is the first indication for a surge in liver transplants in childhood and adolescence in the near future [6,7,8,9,10,11]. 

Non-alcoholic fatty liver disease is diagnosed when >5% of the hepatocytes (Heps) in the liver exhibit fat accumulation (steatosis), assessed by either histology or imaging. Indispensable for the diagnosis of NAFLD are the absence of alcohol consumption, other liver disease etiologies (e.g., viral hepatitis) and use of medications that induce steatosis (e.g., amiodarone). The more aggressive form of NAFLD is characterized by the presence of Hep ballooning and inflammation named non-alcoholic steatohepatitis (NASH). The estimated prevalence of NAFLD patients who develop NASH varies from 63% in Asia to 69% in Europe, but the risk factor remains uninvestigated [3]. The number of NASH cases is projected to increase from 16.52 million cases in 2015 to 27.00 million in 2030 [12]. 

The presence of advanced fibrosis in NAFLD and NASH patients is the most telling marker of mortality and liver-specific morbidity [13,14]. The progression of fibrosis and cirrhosis are caused by uncontrolled extracellular matrix deposition caused mainly by the hepatic stellate cells (HSCs). These cells undergo activation in condition of hepatocellular injury [15]. The NAFLD/NASH patients with high fibrosis stage score are at higher risk of developing hepatic and extra-hepatic malignancies, such as hepatocellular carcinoma (HCC), colorectal and breast cancer then patients with lower stage [16,17]. To date, no pharmacological treatment is available for NAFLD and NASH and the need for an effective therapeutic agent is high [5]. Lifestyle interventions, such as strict calorie-intake control and increase in physical activity, remain the most effective strategies in containment and resolution of NAFLD. 

**Figure 2:**
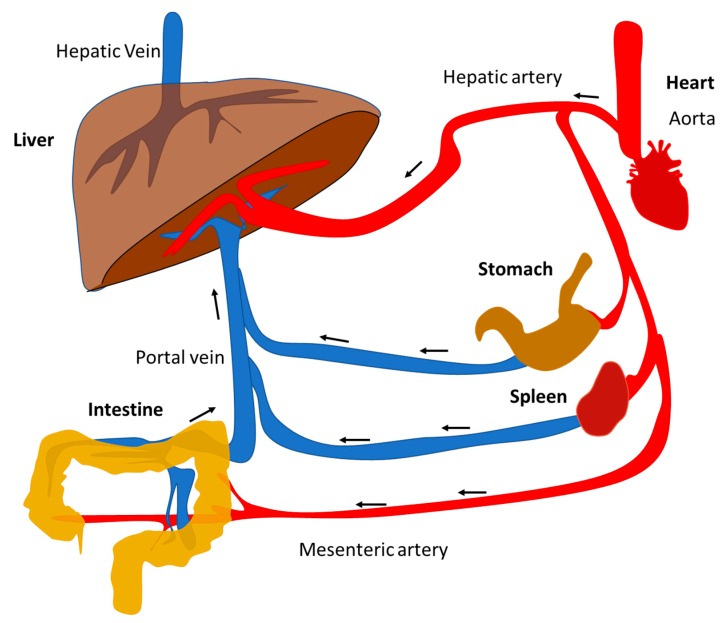
Schematic representation of liver blood circulation.

The liver requires a great amount of oxygen-supplying blood flow to function. The hepatic artery, a ramification from the aorta coming from the heart, provides only the 25% of the necessary blood to the liver. The remaining 75% comes from portal vein, which collects blood from the superior and inferior mesenteric, splenic, gastric, and cystic veins (Figure 2). The strategic position of the liver and its blood supply system makes it the first line of defense of the body by transforming exogenous toxic compounds, such as alcohol and medications, into harmless substances. 

Further, the liver neutralizes a wide range of internal toxic wastes from metabolized nutrients and hormones. These compounds are transformed into water-soluble compounds that can be excreted with bile, stool and urine. The liver has an exceptional ability to synchronize multiple metabolic pathways to maintain the body homeostasis. One of these compensatory capabilities is to switch between fuel sources according to energy needs. For example, the liver stores the nutrients such as glycogen from glucose to then use it when the body plasma glucose level decrease. In case of prolonged absence of glucose, the liver can produce it from a wide range of carbon sources such as glycerol, fatty acids (FAs), lactate and even amino acids (AAs). In excess of sugar or ethanol intake, the liver converts these into smaller, denser compounds known as triglycerides (TG) that the body uses when necessary. This capacity is compromised or even lost in patients with NAFLD. In fact, an unbalanced diet inevitably leads to an “elective malnutrition” resulting from voluntary massive intake of only few categories of macronutrients (e.g., sugar and fat), and little else (e.g., vegetable fibers and vitamins).

In this review, the role of the liver in orchestrating the metabolism in both healthy and non-alcoholic fatty liver (NAFL) conditions will be discussed. A special focus will be given on the impact of protein metabolism to the progression of the disease. 

## 2. Pathobiology of NAFLD

As a progressive disease, NAFLD was initially theorized to have a “two-hit” mechanism, beginning with the uncontrolled fat accumulation in the liver cells, leading to the second metabolic insult that triggers inflammation and fibrosis. Nowadays, evidence support a more complex process involving multiple parallel metabolic hits that promote disease progression, where diet and sedentary lifestyle play a key role in the development of NAFLD [18].

Steatosis is a pathological feature of NAFLD. It is considered when 5% of total Heps contain lipid droplets. The size and distribution of the lipid droplets can distinguish three types of steatosis: Macro, medio and microvesicular [19]. General hypothesis is that small lipid droplets-formed by neutral lipid core such as triglyceride surrounded by a phospholipid monolayer-fuse themselves into a bigger one (macrosteatosis) which gradually disappear with the appearance of fibrosis. Several studies demonstrate that patches of microvesicular steatosis are associated with mitochondria dysfunction, cytoskeleton damage and fibrosis whereas macrosteatosis is deemed to be a benign lesion [20,21,22,23]. This process is elegantly summarized elsewhere [24].

**Figure 3:**
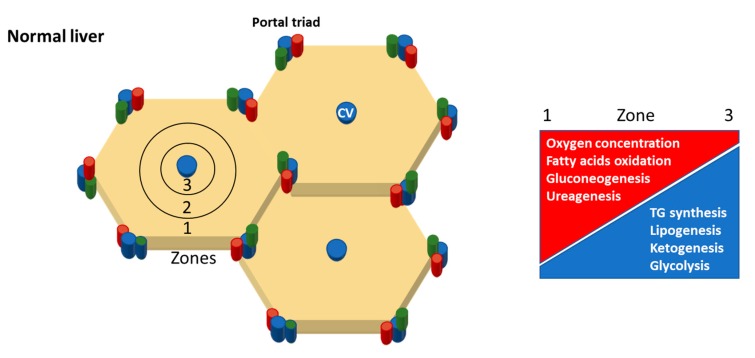
Upper panel, the elementary unit of the liver is called lobule. Central vein (CV, blue) is located in the middle of it and portal triad (hepatic artery, red—portal vein, blue—bile duct, green) at periphery. Three zones can be distinguished. Zone 1, periportal zone; zone 2, the intermediary zone; zone 3 the pericentral zone. Lower panel shows the zonal distribution of the main metabolic processes.

At the histological level, the anatomy of the liver is simpler than that from other organs; it does not have functional areas such as outer cortex and inner medulla of kidney or hypothalamus of the brain. The smallest unit of the liver is called hepatic lobule. It is a hexagonal structure with central vein (CV) in the center and portal triad -hepatic artery-portal vein-bile duct-at the periphery (Figure 3). The CV is characterized by little or no connective tissue allowing free blood plasma exchange with Heps trough specialized liver sinusoidal endothelial cells (LSECs). The Heps have three different membrane domains: Apical (canalicular), lateral and basal (sinusoidal) according to their respective functions. These cells spread out towards the portal triad surrounded at each side by LSECs. The space between the Heps and the LSECs is known as the “space of Disse”; it represents a niche for other liver cell populations such as HSCs and Kupffer cells (KCs). The “stage” where all these actors play is known as “sinusoid” full of nutrient and oxygen rich blood from portal vein and hepatic artery direct towards CV (low O_2_ area) and systemic circulation.

This spatial organization mirrors the heterogeneous enzyme distribution known as metabolic zonation [25]. Portal triad (zone 1) is characterized by high level of gluconeogenesis, fatty acids oxidation and urea synthesis enzymes while CV (zone 3) with high level of enzymes for glycolysis, lipogenesis and De novo lipogenesis (DNL), ketogenesis, protein and xenobiotic metabolism (Figure 3) [26]. 

During the initial phase of the NAFLD, the steatosis is localized around zone 3, where Heps, arranged radially, are more susceptible to fat damage and mitochondrial dysfunction. In more severe form of the disease, the steatosis spreads out all over the liver with an irregular pattern, affecting the organ’s performance and function [27]. While the peri-central fat accumulation is the most common pattern found in adults, azonal, homogenous, as well as peri-portal fat distribution have been described in children [27,28]. 

The Heps close to the periportal area undergo a metabolic adaptation to address the changes in microenvironment composition. They are more resistant to toxic insults and quickly adjust their enzymatic repertoire according to metabolic demands. This ability decreases in the cells close to the peri-central area [29]. These Heps have the highest lipogenic capacity among the non-adipocyte cell in the body. However, the continue exposure to mono and disaccharide along with high fat regimen leads to heps death and loss of zonation. Indeed, both in vivo and in vitro, Heps showed enhanced TG accumulation when glucose or fructose are administered in conjunction with high fat diet compared with only fat supplementation [30]. 

More studies have confirmed this synergistic effect, adding that the most dramatic intrahepatic accumulation of triglycerides is observed when extra calories are introduced between the meals (e.g., snack) [31,32,33]. One additional point supporting these observations is that snacks and sweetened beverages do not contribute to the sense of satiety as solid food underlining that frequency of the meals is more harmful than the meals size [34]. 

## 3. Liver Metabolic Plasticity for Carbohydrates, Lipids and Protein

**Figure 4:**
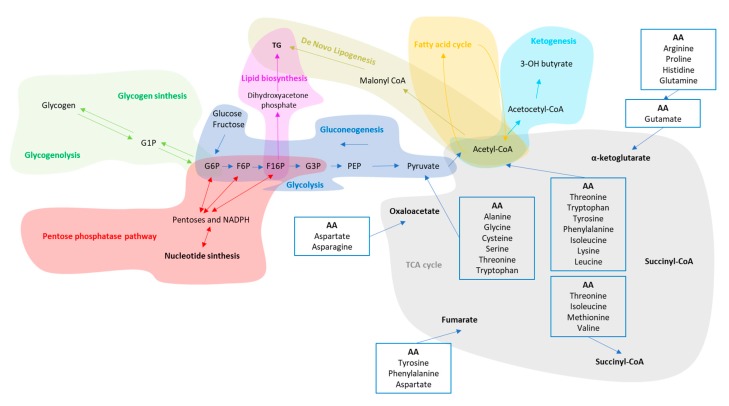
Common intermediaries shared between the main metabolic pathways. In alphabetic order: F16P, Fructose-1,6-Phosphate; F6P, Fructose-6-Phosphate; G1P, Glucose-1-Phosphate; G3P, Glyceraldehyde-3-phosphate; G6P, Glucose-6-Phosphate; NADPH, Nicotinamide adenine dinucleotide phosphate; PEP, Phosphoenolpyruvate.

To survive, an organism needs to convert energy to fuel all the cellular functions. This process is called metabolism and has four goals: (i) energy extraction from nutrients (i.e., carbohydrates (CHO), FAs and protein–(PO)); (ii) synthesis of nucleic acids; (iii) energy storage; (iv) excretion of waste products-mainly nitrogenous compounds. In humans, the achievement of these four goals is accomplished by: (a) glycolysis; (b) gluconeogenesis; (c) pentose phosphate pathway; (d) ketogenesis; (e) FA synthesis and β oxidation; (f) DNL; (g) tricarboxylic acid cycle; (h) amino acid degradation and urea cycle. In healthy organisms, these pathways interact with each other via multiple common metabolic intermediaries. Each pathway is finely regulated through direct action of hormones such as insulin and the body homeostasis is kept at minimum energy cost by the central nervous system (Figure 3). In this paragraph, a quick look to main pathways involved in obtaining energy from food and their intermediates will be given (Figure 4). 

The most primitive mechanism evolved by human cells to extract energy from the transformation of glucose into pyruvate is called glycolysis. It takes place in the mitochondria of almost every organ. When in excess, glucose is stored as glycogen in the liver and skeletal muscle. When glycolysis is activated, the synthesis of new molecules of glycogen is deactivated and the other way around according to fed/starvation state (presence or absence of Adenosine triphosphate, ATP). However, organs such as brain utilize glucose as their preferred or only metabolic fuel supply. Gluconeogenesis is the process that provides glucose to these tissues from precursors such as glycerol, lactate, pyruvate, and glucogenic amino acids; it takes place primarily in the liver and in a specific area of renal cortex [35].

Four intermediaries of the glycolysis glucose-6-phosphate (G6P), fructose-1, 6-phosphate (F16P), fructose-6-phosphate (F6P) and glyceraldehyde-3-phosphate (G3P), can also enter in a parallel pathway of glycolysis known as pentose phosphate pathway (PPP). The primary goal of this pathway is to produce NADPH and ribose 5-phosphate essentials for nucleic acid synthesis and cell replication. *Vice versa*, in the non-oxidative branch of PPP these intermediaries can flow back into glycolysis or gluconeogenesis pathways with a process as known as recycling PPP [36] (Figure 4). 

The liver is the primary location for ketone body synthesis, a process that provides oxidable carbon source from fatty acids and amino acids (especially leucine). This process save glycogen and gluconeogenic skeletal muscle proteins during high energy demand period (e.g., physical activity) and/or absence of glucose [37]. The Ketone bodies increase in period of fasting, starvation and post-exercise. The Heps cannot metabolize the ketone bodies that they produce, therefore the neosynthesized ketone bodies have other extrahepatic non-oxidative metabolic fates. For example, they can be transformed in mitochondrial acetyl-CoA to enter into the TCA cycle for terminal oxidation, enter into cytoplasmic lipogenesis and cholesterol synthesis or excreted with urine [38,39,40]. In the heart, brain and skeletal muscles of healthy humans, ketone bodies represent an essential energetic fuel source (Figure 4).

The liver has an active role in lipids homeostasis intended as TG, cholesterol and its esters, glycolipid and phospholipid metabolism. It exerts a tight control over their utilization as energy substrate (Fatty acid oxidation, FAO inside of the mitochondria), synthesis (FAS, inside the cytoplasm), redistribution and storage into other tissues [41,42]. The level of fat in the blood is generally low under physiological condition even after meals containing over 100 g of fat per day. This is possible thanks to an efficient lipoprotein pathway which transfers dietary lipids directly into the skeletal muscle and adipose tissues packed in chylomicrons, dodging the liver. Different chylomicrons, called remnants because rich in free fatty acids and cholesterol, reach the liver from intestine and are released by skeletal muscle and adipose tissues. Their dimensions and compositions are correlated with the amount of fat in the diet. 

The fatty acids are oxidized in the mitochondria providing ATP and acetyl-CoA. This latter molecule and its derivatives participate in many pathways such as TCA cycle, ketone body formation, cholesterol synthesis, DNL and amino acids metabolism. To note, the substrate for the synthesis of TG are also Glycerol 3-phosphate (G-3-P) and fatty acyl-coenzyme, (fa-CoA) strictly related to the presence of either glucose or pyruvate as substrates. 

Glycerol kinase is a phosphotransferase enzyme crucial for triglycerides and glycerophospholipids synthesis. It catalyzes the transfer of a phosphate from ATP from glycerol to form glycerol 3-phosphate (intermediary of glycolysis). This enzyme has high activity in the liver but low activity in adipose tissue and skeletal muscle contributing to metabolic plasticity of the liver [43,44] (Figure 4). 

*De Novo* Lipogenesis is an important mechanism evolved in the liver to compress high amount of CHO in few molecules of TG and store it in specialized cells called adipocytes. It is suppressed by fasting [45] but activated by high amount of CHO ingested [46,47]. Generally, in lean subjects the contribution of DNL in the energy balance is very low [45,48] (Figure 4). 

Proteins are short and long series of AAs and are traditionally classified as essential and non-essential AAs according to the ability of the organism to synthetize them (Figure 4). The small intestine and liver are the main sites for AAs catabolism, regulating their access to the portal and systemic bloodstreams [49,50]. 

The AAs play a vital role in many biological processes due to many metabolic entry points (Figure 4). As very versatile molecules, AAs can be converted in FAs (short and long chain), glucose, ketone bodies, urea, polyamines, CO_2_ and ammonia but with less energetic efficiency compared with CHO and Fat [51,52,53,54,55]. Most of AAs are metabolized in the liver and kidney, where carbon can contribute to gluconeogenesis and nitrogen waste excreted trough urine. A subgroup of AAs called Branched-Chain AAs (BCAAs) are only oxidized in extra-hepatic tissue such as hearth and skeletal muscle (SM) tissue although the meaning of this different metabolic sites is still not known. However, BCAAs enhance glucose transportation and activate the insulin secretion [56,57]. The ratio of branched-chain amino acids (BCAAs: Valine, leucine, isoleucine) to aromatic amino acids (AAAs: Tyrosine, phenylalanine) has been proposed as a diagnostic marker for assessing liver metabolism and the severity of liver dysfunction [58,59].

The synchronization of all these processes allow the body to keep the energy level constant regardless to the energy source or diet regimens. However, the quantity and frequency of calories intake slowly but inevitably affect this ability as it is the case of NAFLD. 

## 4. Fuel Selection in NAFLD: Evidences and False Myths

As omnivores humans have adapted to extract energy from a variety of sources such as CHO, FA and PO. This ability reached its maximum expression throughout the evolution with fine-tuning of a complicated and interchangeable web of mechanisms that transform, store and redistribute energy to survive to the sudden periods of starvation. Nowadays, this ability has become a disadvantage due to high fat and sugary foods availability and no need for physical activity. The obesogenic environment so created is even more exasperated by modern food policies, where low-cost of highly processed foods (meats, grain and sugary drinks) cost less than healthy foods such as vegetables, fish and fruits [60]. These types of foods cause a metabolic dysregulation including impaired glycemic control and insulin resistance [61,62]. To note that the presence of lipid in the liver seems to be regulated by factors other than visceral and total fat [63]. Indeed, the chronic imbalance between energy intake and energy expenditure is more dangerous than the increase in the body weight per se [64]. In NAFLD context, the massive amount of calories intake overcome the natural adaptation of the body to neutralize the excess of the foods ingestion with loss of control over the metabolism (Table 1).

Two are the major drivers of hepatic steatosis such as FFAs that originate from lipolysis of triglyceride in adipose tissue and DNL. In case of high fat high cholesterol diet, it seems that the most prominent lipids source in NAFLD patients seems to be serum non-esterified fatty acids (NEFA) followed by deactivation of DNL and only small contribution (15%) from dietary fatty acids [81,82,83]. In case of diet based on excess of “sugar” and carbohydrates, DNL is the driving mechanism. High CHO diet is associated with increase of plasma TGs [84] and higher energy expenditure compared with high fat diet, probably due to the activation of sympathetic nervous system [85,86]. It was recently demonstrated that reducing the portion of CHO lowers the intrahepatic lipid accumulation in a few weeks, independently from the weight loss [87,88]. Adoption of this low CHO diet reduces liver inflammation and fibrosis on the long term [89]. On top of that, CHO reduction has been shown to have beneficial effect on glycemic control in T2DM patients regardless from the presence or absence of polyunsaturated fatty acid (ω-3) [90,91]. An important aspect seems that reducing the CHO component in the diet induces a more important beneficial effect than reducing fat component. 

In fact, the high carbohydrate intake is associated with increased severity of NAFLD compared with low carbohydrates-high fat regimen [92]. The recently discovered protective effect of the serum cholesterol seems to go beyond the binding and transport of potentially microorganism-harmful amphipathic and hydrophobic molecules [93]. Indeed, it has been showed that hyperlipidemia can protect against pathogen infection [94]. 

Overnutrition associated with an under-exercising regimen lead to the irreversible dysregulation of body metabolism with extremely harmful consequences for the health. The human body has a very helpful metabolic plasticity, which is very important for two aims: (1) utilize different fuel sources depending on their availability assuring the survival of the organism and, (2) to repair or isolate specific pathways when their disruption occur avoiding lower energy production [95,96]. 

## 5. High Protein Diet in NAFLD, Cure or Disease?

The Food and Nutrition Board of the Institute of Medicine suggests that the daily energy intake for an adult has to come from CHO—45–65%, Fat—20–35% and protein—10–35%. This proportionis associated with reduced risk for developing chronic disease while providing the necessary intakes of essential nutrients [97,98]. Recently, many high protein diets have been proposed to decrease energy intake such as Stillman (64%), South beach (39%), Atkins (35%) and Zone (34%), most of them at the expense of CHO [99,100,101,102]. 

The Stillman diet consists of unlimited calories from protein and fat and only 2% from CHO. It is the most severe ketogenic diet where only lean meats (e.g., lamb, veal etc.), fish (haddock, cod etc.), eggs and cheese made with skim milk are allowed. Stay hydrated and multivitamin supplementation are also essential [102]. Aftereffects of the Stillman diet are easy fatigue, lassitude and mild nausea mainly because of the initial fluid loss. The Stillman diet is strongly recommended for short time period especially in middle-aged persons who may have coronary artery disease because of the large amount of animal products ingested [103]. 

South beach diet is based on foods with low glycemic index (GI). This kind of foods are metabolized slowly by the body and induce smaller fluctuations in blood glucose and insulin levels. This diet consists in 3 phases: (i) CHO deprivation; (ii) limited amounts of foods with low GI are introduced; (iii) Normal-sized portions of all foods are encouraged [104]. Although is less restrictive than the Stillman diet, it still generates some safety concerns about the potential ketosis in the first phase and limited array of foods in the second phase.

The Atkins diet is one of the most popular low-CHO diet and does not require calories or portion control. It consists in 4 phases: (i) “induction” where the daily intake of CHO is limited at 20 g or 10% of calories; (ii) “balancing” where some nutrient-rich carbs such as nuts and seeds are introduced without changing the portion of CHO; (iii) “pre-maintenance” phase where some low sugary fruits, starchy vegetables and whole grains are introduced; (iv) “lifetime maintenance” phase where CHO ranges from 40 to 120 g/day. As the previous two diets, the drastic cut of CHO in the early phase can induce headache, weakness and fatigue. Small amount of extra salt, along with vitamins or supplements are recommended. 

In the Zone diet, the PO, FA and CHO caloric ratio is 30/30/40, where low GI foods and monosaturated fat are preferred. The foods are grouped in blocks according to the PO, FA and CHO content. Each main meal consists of 3 to 5 blocks, while snacks contains one zone block. The limitations of this diet are the difficulty to calculate portions, the massive amount of vegetable ingestion and the menus which are not appealing.

Generally, exchanging CHO for PO has been shown to induce weight reduction and decrease risk factors such as TG and cholesterol as well as increase high-density lipoprotein (HDL) and glucose blood levels [84,105,106]. 

Among these macronutrient categories, protein is the main one that contributes to the satiety, followed by carbohydrate and fat and, therefore promote weight loss [107,108,109]. The digestion, absorption, transport and storage of proteins have the higher oxygen demand compared with FAs and CHO [108,110]. In fact, reduction in fat mass more than the weight reduction seems to have a beneficial effect for the health [111]. 

In the last few years many studies started to questioning the effects of animal (e.g., meat and dairy products) and plant (e.g., grains and legumes) proteins intake on risk to develop cardiovascular disease and cancer [112,113]. In a recent meta-analysis proteins intake from plants is associated with a decrease risk of diabetes, whereas animal proteins with an increase of it [114,115]. 

In a large cohort study of USA citizens, people who have large red and processed meat daily intake have high risk of cardiovascular disease [116]. One more study demonstrates that both white and red meat increase high low-density lipoprotein (LDL) cholesterol level compared with vegetable protein source in healthy subjects under the same high saturated fatty acid (SFA) regimen [117]. Interestingly, all the types of meat, included the “canonical” low-fat meat such as chicken and turkey, increase the risk for developing NAFLD [118]. Positive impact on overall health and mortality was also recently demonstrated using FAs such as oleic and palmitic acids from plant-based foods instead from dairy products [119,120].

Animal proteins contain high level of methionine, homocysteine and cysteine whose metabolism produces sulfate (transsulfuration), important fraction of the daily acid load. The liver is the site where the metabolism of these AAs occurs known as one carbon metabolism. Dysregulation of this pathway lead to accumulation of these AAs, especially homocysteine, in the liver and plasma which have been proved to be a risk factor for NAFLD, cerebrovascular disease and fracture [121,122,123]. 

Plant proteins have high level in glutamate and glycine that requires high amount of hydrogen ions to be metabolized alkalinizing the microenvironment [124,125]. Animal protein-based diets contain high levels of glutamine and ornithine which are major precursors for ammoniagenesis compared with vegetarian diets [126]. Ammonia accumulation has been showed to be toxic for the body and its removal improved greatly liver zonation and functionality [127].

Interestingly, in diabetic patients with cirrhosis, diet rich in vegetable proteins ameliorates the plasma glucose level and hormone response to the meal [128]. Beneficial effects of this diet has been observed also on overall clinical and cognitive improvement in cirrhotic patients with hepatic encephalopathy as well as on nitrogen metabolism [129,130].

One more study demonstrated the reduction of alanine aminotransferase after high protein diet (soy) was administered to the NAFLD subjects [131]. Moreover, high animal protein consumption is associated with increase in renal plasma flow and glomerular filtration rate which do not happen with vegetable protein [132,133].

One more study has shown that vegetable diet with low methionine and aromatic amino acids but rich in BCAAs, improved the health of cirrhotic patients with mild portal-systemic encephalopathy, although 20% of the patients experienced hypoglycemia [134]. In fact, BCAAs stimulates insulin production and glucose uptake from liver and SM cells [135]. Furthermore, BCAAs supplementation in obese cirrhotic men reduces the development of HCC and enhances the survival rate [136,137]. Moreover, it potentiates the antitumor effects of sorafenib in patients with advanced HCC [138]. It has been also shown that dietary supplementation with arginine reduces the level of glucose and improves insulin sensitivity in obese patients and diabetic fatty rats [139,140]. 

Acute glycine supplementation improves glucose tolerance, insulin response and oxidative stress in patients with MetS [141,142,143]. The mechanism may rely on increase responsiveness to the insulin of pancreatic β-cells (acting on glycine receptors [142]) as well as protecting against oxidative damage as precursor of glutathione, purine and creatine [122]. Low plasma level of glycine and BCAAs in patients with NAFLD and T2DM are inversely associated with hepatic insulin resistance and risk to develop T2DM [78,120,144]. High plasma level of proline and glutamate are strongly correlated with β-cell disfunction and hyperinsulinemia [145,146]. 

Epidemiological studies demonstrate that high protein intake does not interfere with calcium homeostasis on the short term while it reduces the incidence of bone fracture on the long term [147]. 

Although the diet rich in vegetable proteins and reduced CHO seems to have the most beneficial effect on preventing the development and progression of metabolic disorders, it must include foods from a wide variety of vegetable sources to supply all the essential AAs [148]. 

High protein diet is a valid therapeutic approach to revert NAFLD. However, the protein source and the functional status of the liver need to be considered.

## 6. High Protein Diets Limitation

High protein diet approach has been criticized by public health perspective because of the negative effects on metabolic environment of the colon. Particularly, the elevated amount of nitrosamine and heterocyclic amine in fecal samples and their link with colorectal cancer [149,150]. Furthermore, sulfates and phosphate coming from the protein metabolism impact greatly on the acid-base balance with deleterious effects on urinary calcium loss, which may promote osteoporosis. [151,152]. High level of AAs in the diet induces augmented glomerular filtration rate, albuminuria, serum uric acid, and urinary pH values which may accelerate chronic kidney disease progression [153].

High protein diet intake derived mainly from dairy products, with high load of glutamic acid and proline, has been associated with high risk to develop diabetes. It has been also shown that high intake of BCAAs in combination with high fat diet, led to accumulation of succinyl and propionyl-CoA which might interfere with glycolysis, FAO, TCA cycle and insulin sensitivity trough mitochondrial stress [154]. 

People adhering to high-protein diets tend to regain weight as soon as the provision of the food stopped, although they had overall health improvement and waist circumferences reduction [155,156]. Recent evidences demonstrated that amino acids overload could lead to the inhibition of insulin signaling and/or insulin resistance through mTOR activation [157,158]. The effects of the high protein diet in patients with NAFLD remain controversial. There is a need of more focused randomized clinical trials where the impact of single amino acids pool contribution on health must be investigated.

## 7. Skeletal Muscle

Skeletal muscle is the largest reservoir of both proteins and AAs with a mass around the 40–45% of body weight. It is also the primary tissue participating to the total-body insulin-mediated glucose disposal. In the last decade, many groups demonstrated incontrovertibly the participation of this tissue to the progression of NAFLD. In this section, loss of SM as well as its participation to the synthesis of TG will be discussed. 

Sarcopenia is a gradual loss of skeletal muscle tissue due to reduced protein synthesis with functional decline and poor quality of life. In adults, the loss of skeletal muscle mass proceeds at 0.5%/year rate, however it accelerates over age 65 or with deficiency in essential AAs [159,160]. In chronic diseases, SM loss (wasting) can be associated with impaired contractile function and strength defined as myopenia and dynapenia, respectively [161,162]. In obese individuals, the presence of sarcopenia leads to a far worse dysglycemia and insulin resistance due to inter-and intra-muscular fat deposits [163]. 

The accumulation of inter and intra-muscular fat is associated with sarcopenia [164]. This process is known as myosteatosis and increase with adiposity and age. New evidences demonstrate that SM participate to the synthesis and release of TG when sucrose diet was administered [165]. The muscular TG production is also associated with increased mortality in cirrhotic patients [166]. 

Loss of muscle mass is associated with proteolysis and reduction of ammonia removal from blood circulation. The SM tissue is also the main organ-target for insulin. Muscle protein breakdown contributes to the accumulation of acetyl-CoA which in turn inhibit the glycolysis and FAO, especially in a continuously secreted insulin regimen. The hampering of these the two latter pathways exacerbates IR in the liver contributing to the progression of the disease. 

Chronic liver damage and cell death are strictly correlated with sarcopenia and often cause of increase morbidity and mortality in cirrhotic patients [167]. Even after liver transplantation in those patients, metabolic blood parameters return within the normal range, increased body weight is observed, but sarcopenia does not revert it [168,169]. Further, loss of SM is associated with longer hospital stay and increased risk of infection along with malnutrition [170,171]. 

The preservation of SM mass is crucial for the development of NAFLD. Different therapeutic strategies have been identified to reduce SM loss and improve insulin sensitivity and glucose homeostasis. Sarcopenia can be partially attenuated by BCAAs supplementation, which reduce protein catabolism and lower systemic ammonia with positive effect on the survival rate of cirrhotic patients [172,173,174]. Further, the supplementation of potassium bicarbonate has been shown to preserve muscular mass during aging by reducing nitrogen excretion. Moreover, dairy products, grains, and meats results in excess acid in the body which in turn stimulates the breakdown of muscle. Neutralizing acidity using potassium bicarbonate reduces muscle mass loss [175,176]. 

On the other hand, the oral administration of BCAAs seems to improve the glucose uptake by SM cells ameliorating NASH features [177]. Another evidence is that the supplementation of arginine lower plasma TGs and glucose, promoting SM tissue mass over fat gain in diet-induced obese and NASH animal models [178,179]. 

In obese subject, weight loss is associated with increase in oxidative capacity and capillarity of SM tissue compared with when the weight is gained [180]. Another study confirms that a short period of fasting increase oxygenation level and decrease protein breakdown in overweight subjects [181]. The switch from glycolytic to fat oxidation pathway for energy production might be behind the increase of muscle work efficiency in people losing weight [69,182,183]. 

These studies demonstrate that the SM mass play a crucial role in the progression as well as in the treatment of NAFLD. 

## 8. Physical Activity

Compared to our ancestors, the estimated energy expenditure per kilogram of body is <40% due the easily available daily caloric load [184]. The lack of PA along with high frequency of junk food meals and sedentary lifestyle are the main causes for the epidemic obesity in western countries [31,185]. In addition, weight gain results in decreased muscle work efficiency associated with a faster poor outcome [69]. Physical activity induces a body transition from fat loss to muscle gain. Many obese patients experience improvement in liver histology without significative weight loss [186,187,188]. However, few studies addressed the question of which type of PA has a positive impact on NAFLD feature and the most effective method for that is still matter of debate.

Two out of three cross-sectional studies demonstrated a strong correlation between cardiorespiratory fitness (in terms of VO_2,max_) with accumulation of the fat within the liver [63,189,190]. More studies confirmed the association between low fitness with high liver fat and mortality, which is independent from abdominal and total fat [191,192]. Interestingly, fitness greatly reduces the lipid content in the liver independently from weight loss [193]. 

Lipid oxidation sources during exercise are plasma free FAs, plasma VLDL-TG-derived fatty acids and intramyocellular triacylglycerol (IMTG). During submaximal exercise, SM tissue takes up around 65% of the FAs from blood circulation and directly oxidized it [194,195] whereas only around 3% comes from circulating very low-density lipoprotein-TG (from liver) [196]. Interestingly, when adipose tissue lipolysis is inhibited during exercise, plasma free fatty acids decrease but muscle TG content and glycogen oxidation rates increase [197,198].

Most of the changes in circulating lipids uptake and oxidation take place the day after a single and intense session of training [199,200] whereas a 40% decrease in IMTG content was detected after 45 min to 2 h of moderate-intensity training [201]. Furthermore, regular and moderate aerobic training lower the level of plasma TG and increase the hepatic protein synthesis [202]. BCAAs are metabolized mainly in SM tissue due to high level of the first enzyme involved in the BCAA catabolism pathway known as branched-chain-amino-acid aminotransferase. This subgroup of AAs seems to exert stimulatory effect on the anabolic and an inhibitory effect on catabolic processes as well as attenuates SM damage in resistance-trained individuals [203]. The underlying mechanisms is still not known but the BCAA supplementation of the diet is a popular practice among professional and recreational exercisers and athletes [203].

## 9. Conclusions

Metabolic syndrome is a chronic disease that extends over decades, inducing metabolic alterations and changes in body composition. The body, as all the biological systems, adapts itself to diet changes as long it is possible. Unfortunately, the lack of time and information together with low cost made the people of industrialized countries rely on a diet based on high-processed foods, especially from animal sources. 

Meat and dairy products are identified to have better digestibility and higher protein to energy ratios compared with plant proteins. While animal proteins are rich in iron, zinc and vitamin B12, plant proteins are rich in magnesium, fiber and vitamin E. However, in the last decade, the people are pushed to eat the same highly processed repertoire of foods repeatedly leading to different alimentary deficiency such as vitamins and fiber. 

High protein hypo-energetic diet, especially from vegetable origin, with low CHO and sugar would be a promising therapeutic strategy to revert NAFLD phenotype and reduce insulin resistance, when liver function and muscle catabolism are not compromised [54,204,205]. In addition, moderate increase in physical activity trough repetitive sessions with moderate intensity and duration plus supplementation with BCAAs seem to alleviate sarcopenia delaying the development of obesity. 

An equilibrate diet, with 80% of the proteins coming from vegetable source, along with PA offers a good healthy lifestyle to delay T2DM and HCC while waiting more stronger data on pharmacological treatments. 

## Figures and Tables

**Table 1:** Summary of the impact of intrahepatic lipid accumulation on main metabolic pathways.

	Compartment/Organ	Health	NAFLD
Glycolysis /gluconeogenesis	Cytosol-all organs	Removal of Excess of glucose in the blood after meals trough glucose oxidation and glycogen storage in the liver and muscle. The liver is also able to release glucose in the blood during fasting trough glycogenolysis and gluconeogenesis to avoid hypoglycemic events.	Liver and muscle cells become insulin resistant. In the liver, hepatocytes increase the production rate of glycogenolysis and gluconeogenesis as well as cholesterol and triglyceride synthesis [65,66,67]. The skeletal muscle cells decrease blood glucose uptake and their work efficiency [68,69].
Pentose phosphate pathway (PPP)	Cytosol-Liver, mammary gland and adrenal cortex.	The PPP generates either the ribose 5-phosphate, one of the precursors for the synthesis of nucleotides and erythrose-4-phosphate used in the synthesis of aromatic amino acids.	Hepatic PPP increases in parallel with lipogenesis [70].
Ketogenesis (Kt)	Mitochondria-Liver	The Ketogenesis breakdown ketogenic amino acids and fatty acids under fasting or caloric restriction conditions.	Obesogenic diets diminish the free fatty acid-induced ketogenesis according to the stage of the disease [71,72,73].
Fatty acid synthesis (FAs)/β oxidation (β-Ox)	Cytoplasm/Mitochondria-Liver and adipose tissue	The FAs uses the end product of glucose metabolism, the acetyl-CoA, and convert it to fatty acids for the synthesis of cellular membranes, energy storage, and intracellular signaling pathways. Acetyl-CoA can be also esterified with glycerol to form triacylglycerol, packed in VLDL and secreted from the liver. With β-Ox, fatty acids molecules are used to generate acetyl-CoA.	IR increases lipolysis from peripheral adipose tissue as well as adipose-derived NEFA influx to the liver [74]. In addition, β-Ox is impaired due to mitochondrial dysfunction [24].
De Novo Lipogenesis (DNL)	Cytosol-Liver	DNL synthetizes FA from acetyl-CoA produced when glycolysis is increased. DNL is suppressed by fasting [48].	IR induces an increase in DNL which contribute to synthesis and accumulation of TG in the liver [75,76].
Citric Acid Cycle (TCA)	Mitochondria-all organs	The TCA oxidize amino acids, fatty acids, and carbohydrates to provide most of the energy used by cells in presence of oxygen.	Lipids overload induce increase in hepatic mitochondrial oxidative and anaplerotic TCA cycle activity [73,77].
Amino acid degradation and Urea Cycle	Cytosol/mitochondria-Small intestine, liver, kidney and skeletal muscle.	Amino acids are precursors for the synthesis of a variety of molecules vital to the health, growth, development, reproduction, and homeostasis of the organism.	Intrahepatic fat accumulation induces increase of amino acids in plasma, especially for the branched ones which correlates with more liver damage [78,79]. In addition, progressive deactivation of urea cycle take place with subsequent ammonia accumulation and progression of liver disease [80] as well as loss of muscle mass.

NAFLD: Non-alcoholic fatty liver disease.

## Abbreviations

NAFLD	Non-alcoholic fatty liver disease
AAAs	Aromatic amino acids
AAs	Amino acids
ATP	Adenosine triphosphate
BCAAs	Branched-Chain amino acids
CHO	Carbohydrates
CV	Central vein
DNL	*De novo* lipogenesis
F1,6P	Fructose-1, 6-phosphate
F6P	Fructose-6-phosphate
fa-CoA	Fatty acyl-coenzyme
FAO	Fatty acid oxidation
FAs	Fatty acids
FAS	Fatty acid synthesis
G3P	Glyceraldehyde-3-phosphate
G-3-P	Glycerol 3-phosphate
G6P	Glucose-6-phosphate
HCC	Hepatocellular carcinoma
HDL	High-density lipoprotein
Heps	Hepatocytes
HSCs	Hepatic stellate cells
IMTG	Intramyocellular triacylglycerol
KCs	Kupffer cells
LDL	Low-density lipoprotein
LSEC	Liver sinusoidal endothelial cells
MetS	Metabolic syndrome
NASH	Non-alcoholic steatohepatitis
NEFA	Non-esterified fatty acids
PO	Proteins
PPP	Pentose phosphate pathway
SFA	Saturated fatty acid
SM	Skeletal muscle
T2DM	Diabetes mellitus type 2
TG	Triglycerides
